# Synthesis and Biological Evaluation of Pyrazolo[1,5-a]-pyrimidine-containing ^99m^Tc Nitrido Radiopharmaceuticals as Imaging Agents for Tumors

**DOI:** 10.3390/molecules15128723

**Published:** 2010-11-30

**Authors:** Rui Ding, Yong He, Jingli Xu, Hang Liu, Xiao Wang, Man Feng, Chuanmin Qi, Junbo Zhang

**Affiliations:** Key Laboratory of Radiopharmaceuticals, College of Chemistry, Beijing Normal University, Beijing 100875, China; E-Mails: dingrui@mail.bnu.edu.cn (R.D.); heyong@mail.bnu.edu.cn (Y.H.); xujingli@mail.bnu.edu.cn (J.X.); liuhang@mail.bnu.edu.cn (H.L.); wangxiao@mail.bnu.edu.cn (X.W.); fengman@mail.bnu.edu.cn (M.F.); zhjunbo@bnu.edu.cn(J.Z.).

**Keywords:** pyrazolo[1,5-a]pyrimidine, tumor imaging, [^99m^TcN]^2+^ complex

## Abstract

The compound 5-((2-aminoethylamino)methyl)-7-(4-bromoanilino)-3-cyano-pyrazolo[1,5-a]pyrimidine (ABCPP) was synthesized and conjugated with *N*-mercapto-acetylglycine (MAG), *N*-mercaptoacetylphenylalanine (MAF) and *N*-mercaptoacetylvaline (MAA), respectively. These three compounds were labeled successfully with [^99m^TcN]^2+^ intermediate in high radiochemical purities. Biodistribution in tumor-bearing mice demonstrated that the three new complexes showed tumor accumulation, high tumor-to-muscle (T/M) ratios and fast clearance from blood and muscle. Among them, the ^99m^TcN-MAG-ABCPP showed the most favorable characteristics, with tumor/blood and tumor/ muscle ratios reaching 1.51 and 2.97 at 30 min post-injection, 1.84 and 2.49 at 60 min post-injection, suggesting it could be further studied as potential tumor imaging agent for single photon emission computed tomography (SPECT).

## 1. Introduction 

Tumors are among the most common causes of death in the World. Recently, the use of pyrazolo[1,5-a]pyrimidine derivatives as antitumor agents for the treatment of cancer has come under much attention in a growing number of therapeutic areas [1,2,3,4,5,6]. Among them, 3-cyano-5,7-disubstistituted pyrazolo[1,5-a]pyrimidine derivatives showed significant antitumor properties [7,8].

The novel nitrido core [^99m^TcN]^2+^ is isoelectronic with the [^99m^TcO]^3+^ core. The nitrido ligand is a powerful π-electron donor and shows a high capacity to stabilize the Tc(V) oxidation state [9]. The [^99m^TcN]^2+^ core exhibits a very high chemical stability over a wide range of experimental conditions (such as oxidation-reduction reactions and pH variations), and high affinity toward chelating ligands containing sulfur atoms. The presence of the [^99m^TcN]^2+^ core in the molecular structure of a radiopharmaceutical may dramatically affect its physical and biological behaviour [10]. A convenient preparation of ^99m^Tc nitrido complexes at the tracer level and in sterile and pyrogen-free conditions in which an intermediate ^99m^TcN complex of succinic dihydrazide (SDH) is initially formed in the presence of stannous chloride as reducing agent, has been extensively investigated [11]. So far, the use of the 3-cyano-5,7-disubstistituted pyrazolo[1,5-a]pyrimidine moiety in the preparation of ^99m^TcN complexes as targeted agents for tumor imaging has not been reported. Therefore, it may be of great interest to probe the ^99m^Tc nitrido chemistry with some ligands involving the ABCPP molecular structure to explore the biological behaviour of a potential new class of diagnostic agents. 

The purpose of this study was to conjugate ABCPP with three chelating agents (MAG, MAF and MAA), and to evaluate the feasibility of the ^99m^Tc-labeled 3-cyanopyrazolo[1,5-a]pyrimidine derivatives as useful candidates for tumor imaging.

## 2. Results and Discussion 

### 2.1. Chemistry

**Scheme 1:**
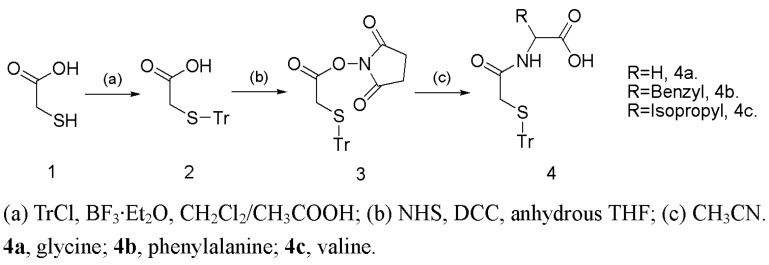
Synthesis of Tr-MAG, Tr-MAF and Tr-MAA.

The preparation of *N*-[(Tritylmercapto)acetyl]glycine (Tr-MAG, **4a**) was carried out using the procedure shown in Scheme 1. After protecting the thiol group with trityl chloride, the resulting compound **2** was reacted with *N*-hydroxysuccinimide (NHS) using dicyclohexylcarbodiimide (DCC) as condensation reagent to obtain the active ester **3**. The active ester **3** was reacted with the amine group of glycine to provide the Tr-MAG (**4a**). *N*-[(Tritylmercapto)acetyl]phenylalanine (Tr-MAF, **4b**) and *N*-[(tritylmercapto)acetyl]valine (Tr-MAA, **4c**) were synthesized by the same procedure as **4a**.

**Scheme 2:**
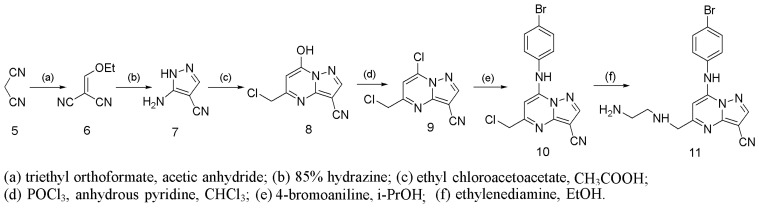
Synthesis of ABCPP.

The synthesis of 5-((2-aminoethylamino)methyl)-7-(4-bromoanilino)-3-cyano-pyrazolo[1,5-a]-pyrimidine (ABCPP) was performed according to the procedure outlined in Scheme 2. Malononitrile (**5**) was reacted with triethyl orthoformate in acetic anhydride to give the intermediate **6**. Treatment with hydrazine generated compound **7**. Cyclization of **7** with ethyl chloroacetoacetate gave the intermediate **8**. Chlorination of **8** with phosphoryl trichloride in the presence of anhydrous pyridine gave the key intermediate **9 [12,13]**. Substitution of 7-Cl in **9** with 4-bromoaniline provided the product **10**. Treatment of **10** with ethylenediamine in ethanol generated the desired compound **11**.

**Scheme 3:**
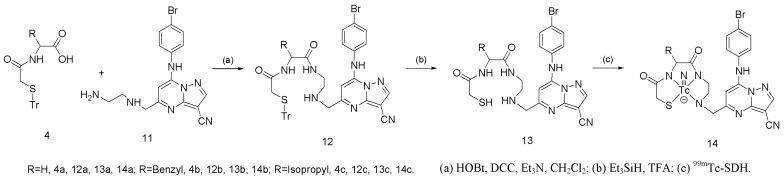
Synthesis of ^99m^TcN-MAG-ABCPP, ^99m^TcN-MAF-ABCPP and ^99m^TcN-MAA-ABCPP.

The compound Tr-MAG-ABCPP was synthesized by conjugating Tr-MAG with ABCPP using 1-hydroxybenzotriazole (HOBt) as nucleophilic catalyst and DCC as condensation agent. The reaction is shown schematically in Scheme 3. The thiol group was deprotected in trifluoroacetic acid (TFA) to give **13a**. For labeling, ^99m^TcN-MAG-ABCPP was prepared through a SDH kit. [^99m^TcO_4_]^-^ was reacted with SDH in the presence of stannous chloride as reducing agent to form a technetium-99m nitrido intermediate. The [^99m^TcN]^2+^ is a suitable substrate for the substitution reaction with MAG-ABCPP at 100 ºC for 15 min to give the final complex ^99m^TcN-MAG-ABCPP (**14a**). ^99m^TcN-MAF-ABCPP (**14b**) and ^99m^TcN-MAA-ABCPP (**14c**) were prepared using the same method.

**Figure 1:**
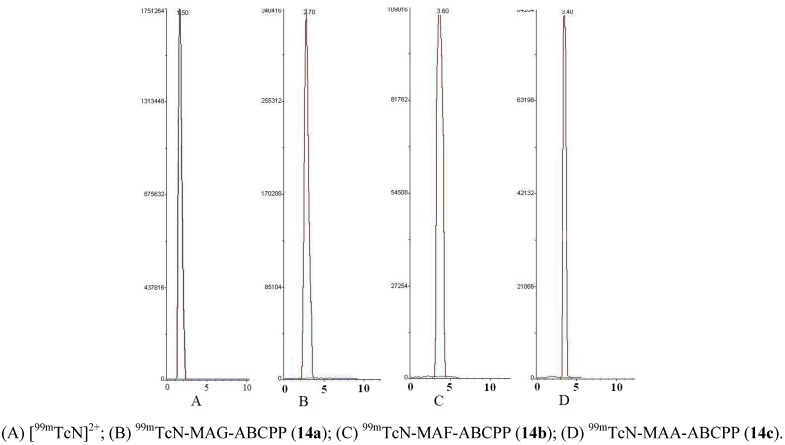
Radio-HPLC chromatograms.

The radiochemical purity of the complexes was routinely checked by HPLC. The HPLC pattern of ^99m^TcN-MAG-ABCPP, ^99m^TcN-MAF-ABCPP and ^99m^TcN-MAA-ABCPP are shown in Figure 1. It was observed that the retention time of [^99m^TcN]^2+^_int_ was 1.5 min, while those of ^99m^TcN-MAG-ABCPP, ^99m^TcN-MAF-ABCPP and ^99m^TcN-MAA-ABCPP were found to be 2.7 min, 3.6 min and 3.4 min, respectively. The radiochemical purity of the three products was over 90% immediately after the preparation.

### 2.2. Measurement of partition coefficients

The partition coefficients were determined by mixing the complex with an equal volume of 1-octanol and phosphate buffer (0.025 mol/L, pH 7.4) in a centrifuge tube. The partition coefficients (logP) of ^99m^TcN-MAG-ABCPP, ^99m^TcN-MAF-ABCPP and ^99m^TcN-MAA-ABCPP are shown in Table 1. All of them were hydrophilic. ^99m^TcN-MAG-ABCPP was the most hydrophilic one and ^99m^TcN-MAA-ABCPP was more hydrophilic than ^99m^TcN-MAF-ABCPP.

### 2.3. In vitro stability study

As the HPLC analysis results for the three complexes indicate, they were all stable in PBS after incubation for 2 h.

### 2.4. Biodistribution study

The biodistribution results are summarized in Table 2, Table 3 and Table 4. There were significant similarities in the biodistribution patterns of these three complexes, which all demonstrated tumor accumulation and high tumor-to-muscle (T/M) ratios. The blood and muscle clearance was faster than that of the tumor so that the tumor/blood and tumor/muscle ratios increased with time. For ^99m^TcN-MAG-ABCPP, the tumor/blood and tumor/muscle ratios reached 1.51 and 2.97 at 30 min post-injection, 1.84 and 2.49 at 60 min post-injection. For ^99m^TcN-MAF-ABCPP, the tumor/blood and tumor/muscle ratios reached 0.51 and 2.56 at 30 min post-injection. For ^99m^TcN-MAA-ABCPP, the tumor/blood and tumor/muscle ratios reached 0.72 and 2.32 at 30 min post-injection. Early hepatic and renal activity reflected the fact that the three complexes were excreted through the hepatobiliary as well as the renal system. The three complexes were hydrophilic, so that they were unable to cross the blood brain barrier, thus making their brain uptake much lower. Among them, ^99m^TcN-MAG-ABCPP showed the most favorable characteristics with highest uptake at the tumor site and fast clearance from blood and muscle.

## 3. Experimental 

### 3.1. General

^99^Mo/^99m^Tc generator was obtained from the China Institute of Atomic Energy (CIAE). All other chemicals were of analytical grade and were used without further purification. ^1^H- and ^13^C-NMR spectra were recorded in CDCl_3_ or DMSO-d_6_ on a Bruker spectrometer operating at 400 and 100 MHz, respectively. The IR spectra were recorded on a Nicolet-AVATAR 360 FT-IR spectrometer using KBr pellets in the 4,000-400 cm^-1^ region. ESI-MS were performed on Waters LCT Premier XE. HPLC analyses were performed on a Shimadzu SCL-10 AVP equipped with a Packard 500 TR series flow scintillation analyzer. A C-18 reversed-phase Alltima column (5um, 150 mm × 4.6 mm) was used for radiochemical purity analysis.

### 3.2. Ligand synthesis

The synthesis of compounds **2** and **3** was carried out by the same procedure described in ref. [14]. The preparation of compounds **6** and **7** was carried out by the same method given in ref. [15] and the preparation of compounds **8** and **9** was carried out by the same methods as in ref. [12] and ref. [13]. The preparation and the analysis data of the other compounds are shown below.

*N-[(Tritylmercapto)acetyl]glycine (Tr-MAG)* (**4a**). Compound **3** (0.30 g, 0.7 mmol) was dissolved in acetonitrile (15 mL). Glycine (0.105 g, 1.4 mmol) in sodium hydroxide (0.2 M, 1 mL) was added dropwise. The reaction solution was stirred for 2 h at 50-60 ºC. Distilled water (1 mL) was added to the reaction mixture and the pH was adjusted to 1. The acetonitrile was removed *in vacuo* and a white solid precipitated. The white residue was recrystallized from ethyl acetate to give compound **4a** (0.22 g, 78%); m.p.: 164-166 ºC; IR (KBr, cm^-1^): ν 3434.4, 3344.0, 2960.3, 2926.5, 1732.4, 1619.0, 1526.7, 1487.9, 1445.3, 1247.6, 1224.1, 1210.7, 740.7, 703.9; ^1^H-NMR (CDCl_3_): δ 3.13 (2H, s), 3.62 (2H, d, *J* = 5.1 Hz), 6.46 (1H, t, *J* = 4.9 Hz), 7.15(3H, m), 7.22 (6H, m), 7.36 (6H, d, *J* = 7.6 Hz); ^13^C-NMR (CDCl_3_): δ 35.4, 41.6, 67.9, 127.1, 128.2, 129.4, 143.8, 169.0, 172.2. 

*N-[(Tritylmercapto)acetyl]phenylalanine (Tr-MAF)* (**4b**). **4b** was obtained with the same method of **4a** using phenylalanine instead of glycine (yield: 59%); m.p.: 128-130 ºC; IR (KBr, cm^-1^): ν 3448.2, 3340.0, 3026.9, 2928.0, 1727.1, 1626.7, 1528.3, 1492.8, 1443.4, 1222.9, 1203.4, 1183.6, 741.9, 700.4; ^1^H-NMR (CDCl_3_): δ 3.07 (4H, m), 4.48 (1H, dm, *J* = 6.2 Hz), 6.54 (1H, d, *J* = 6.2 Hz), 7.10 (2H, m), 7.25 (18H, m); ^13^C-NMR (CDCl_3_): δ 36.0, 37.0, 53.8, 68.1, 127.2, 127.3, 128.2, 128.7, 129.4, 129.5, 135.5, 143.9, 169.1, 173.6.

*N-[(Tritylmercapto)acetyl]valine (Tr-MAA)* (**4c**). **4c** was obtained with the same method of **4a** using valine instead of glycine (yield: 65%); m.p.: 146-148 ºC; IR (KBr, cm^-1^): ν 3433.5, 3339.4, 3055.4, 2960.3, 2928.7, 1738.7, 1720.0, 1635.8, 1534.3, 1488.8, 1441.3, 1422.1, 1380.8, 1304.3, 1273.4, 1213.3, 1081.8, 1035.1, 1000.3, 884.9, 757.5, 738.1, 700.5; ^1^H-NMR (CDCl_3_): δ 0.88 (6H, d, *J* = 6.8 Hz), 2.10 (1H, m), 3.13 (2H, s), 4.31 (1H, dd, *J* = 4.9, 3.2 Hz), 6.62 (1H, d, *J* = 8.2 Hz), 7.22 (3H, m), 7.28 (6H, m), 7.38 (6H, d, *J* = 7.6 Hz); ^13^C-NMR (CDCl_3_): δ 17.9, 18.8, 31.0, 36.2, 57.5, 68.2, 127.2, 128.2, 129.6, 144.0, 168.8, 175.4.

*5-Chloromethyl-7-(4-bromoanilino)-3-cyanopyrazolo[1,5-a]pyrimidine* (**10**). A mixture of **9** (0.8 g, 3.5 mmol), 4-bromoaniline (0.8 g, 4.6 mmol), and isopropanol (10 mL) was heated at 60 ºC for 2 h. After cooling to room temperature, a solid mass precipitated. Recrystallization from methanol gave **10** as a light yellow powder (0.81 g, 66%); m.p.: 206-208 ºC; IR (KBr, cm^-1^): ν 3439.1, 3328.8, 3275.4, 3085.8, 2973.2, 2233.0, 1626.9, 1600.0, 1572.0, 1536.4, 1483.7, 1456.9, 1416.1, 1406.5, 1317.6, 1265.1, 1220.6, 1193.6, 1073.8, 1009.7, 829.9, 780.5, 744.6; ^1^H-NMR (DMSO-d_6_): δ 4.73 (2H, s), 6.67 (1H, s), 7.41 (2H, d, *J* = 8.7 Hz), 7.68 (2H, d, *J* = 8.7 Hz), 8.79 (1H, s), 10.69 (1H, s); ^13^C-NMR (DMSO-d_6_): δ 45.8, 79.5, 89.4, 113.9, 119.0, 127.0, 132.5, 135.7, 146.6, 147.1, 150.6, 160.5.

*5-((2-Aminoethylamino)methyl)-7-(4-bromoanilino)-3-cyanopyrazolo[1,5-a]pyrimidine (ABCPP)* (**11**). A mixture of **10** (0.725 g, 2 mmol), ethylenediamine (0.30 g, 5 mmol), and ethanol (15 mL) was heated to reflux for 4 h. Solvent was removed *in vacuo* to afford the yellow crude product which was recrystallized from methanol to obtain compound **11** (0.56 g, 75%); IR (KBr, cm^-1^): ν 3447.0, 2922.2, 2221.1, 1621.1, 1595.5, 1565.1, 1536.8, 1479.7, 1288.4, 1192.1, 1071.1, 1009.7; ^1^H-NMR (DMSO-d_6_): δ 2.65 (2H, t, *J* = 5.9 Hz), 2.76 (2H, t, *J* = 5.9 Hz), 3.67 (2H, s), 6.33 (1H, s), 7.22 (2H, d, *J* = 8.6 Hz), 7.59 (2H, d, *J* = 8.6 Hz), 8.49 (1H, s); ^13^C-NMR (DMSO-d_6_): δ 46.5, 48.8, 53.9, 77.0, 88.0, 115.1, 115.6, 125.7, 132.0, 142.4, 145.3, 147.1, 151.9, 163.3; MS (ESI): M = 386.2 (Found: 386.3).

*Conjugation of Tr-MAG and ABCPP (Tr-MAG-ABCPP)* (**12a**). A mixture of **11** (386mg, 1mmol), triethylamine (0.21 mL, 1.5 mmol) and anhydrous dichloromethane (8 mL) was stirred at room temperature for 5 min. Then **4a** (407 mg, 1 mmol) and HOBt (150 mg, 1.1 mmol) were added. DCC (230 mg, 1.1 mmol) in 4 mL of anhydrous dichloromethane was added dropwise to the solution at 0 ºC. The mixture was stirred for 30 min at 0 ºC and then overnight at room temperature. White precipitate was removed by filtration. The organic phase was washed with saturated aqueous sodium bicarbonate, brine, dried over anhydrous sodium sulfate, filtered, and concentrated. The crude product was purified by column chromatography (silica gel; petroleum ether-ethyl acetate = 1:5) to afford the desired compound **12a** (220 mg, 29%); m.p.: 185-187 ºC; IR (KBr, cm^-1^): ν 3430.5, 3083.8, 3056.1, 3018.0, 2928.1, 2844.3, 2230.2, 1624.0, 1596.2, 1567.0, 1541.6, 1485.7, 1444.3, 1315.3, 1295.1, 1193.6, 1110.1, 1074.1, 1011.0, 822.5, 743.9, 701.6, 614.9; ^1^H-NMR (CDCl_3_): δ 3.01 (2H, s), 3.14 (2H, s), 3.51 (2H, s), 3.68 (2H, s), 4.22 (2H, s), 6.68 (1H, s), 6.89 (1H, s), 7.18 (11H, m), 7.32 (6H, m), 7.50 (2H, d, *J* = 6.4 Hz), 8.18 (1H, s); ^13^C-NMR (DMSO-d_6_): δ 35.9, 42.4, 45.4, 47.2, 53.6, 65.9, 79.2, 88.5, 114.0, 118.7, 126.5, 126.7, 126.8, 128.0, 129.0, 132.4, 135.9, 144.0, 146.1, 146.9, 150.5, 167.6, 168.9; MS (ESI): M = 759.7 (Found: 759.5).

*Conjugation of Tr-MAF and ABCPP (Tr-MAF-ABCPP)* (**12b**). **12b** was prepared with the same procedure as **12a**; yield: 22%; m.p.: 188-189 ºC; IR (KBr, cm^-1^): ν 3443.9, 3363.6, 3297.4, 3059.9, 3024.0, 2916.3, 2851.0, 2229.8, 1678.9, 1664.1, 1619.5, 1593.2, 1563.7, 1534.9, 1481.4, 1444.9, 1316.1, 1286.9, 1190.1, 1014.3, 830.9, 743.1, 702.0, 672.1, 623.5; ^1^H-NMR (CDCl_3_): δ 2.83 (1H, m), 2.92 (1H, m), 3.02 (2H, s), 3.16 (1H, m), 3.24 (1H, m), 3.48 (2H, m), 3.73 (2H, s), 4.25 (1H, m), 5.89 (1H, t), 6.46 (1H, s), 6.59 (1H, d, *J* = 7.0 Hz), 7.22 (12H, m), 7.28 (4H, m), 7.32 (6H, m), 7.63 (2H, d, *J* = 8.6 Hz), 8.28 (1H, s); ^13^C-NMR (DMSO-d_6_): δ 37.7, 47.5, 47.7, 54.0, 54.2, 65.8, 78.6, 88.1, 114.2, 118.2, 126.1, 126.4, 126.7, 127.9, 128.0, 129.0, 129.1, 132.3, 137.6, 144.0, 146.1, 146.5, 150.8, 165.9, 167.0, 170.5; MS (ESI): M = 849.8 (Found: 849.6).

*Conjugation of Tr-MAA and ABCPP (Tr-MAA-ABCPP)* (**12c**). **12c** was prepared with the same procedure as **12a**; yield: 19%; m.p.: 165-167 ºC; IR (KBr, cm^-1^): ν 3446.2, 3083.8, 3047.9, 2960.8, 2926.1, 2226.1, 1647.4, 1622.3, 1593.1, 1563.6, 1484.3, 1444.7, 1308.8, 1274.2, 1188.0, 1065.2, 1008.2, 802.2, 743.0, 700.6, 621.9; ^1^H-NMR (CDCl_3_): δ 0.85 (6H, dd, *J* = 6.8, 2.2Hz), 2.78 (1H, m), 3.03 (2H, s), 3.35 (4H, m), 3.83 (2H, s), 3.95 (1H, m), 6.29 (1H, t), 6.48 (1H, s), 7.21 (5H, m), 7.28 (6H, m), 7.36 (6H, m), 7.62 (2H, m), 8.28 (1H, s); ^13^C-NMR (DMSO-d_6_): δ 18.1, 19.0, 30.3, 35.9, 38.6, 42.1, 47.8, 54.0, 58.1, 65.9, 78.7, 88.2, 114.2, 118.3, 126.4, 126.7, 128.0, 129.1, 132.4, 144.1, 146.0, 146.6, 150.7, 167.3, 170.6; MS (ESI): M = 801.8 (Found: 801.6).

### 3.3. Radiochemistry

The tritylated compound **12a** (5 mg) was treated with trifluoroacetic acid under cation trapping conditions (5% triethylsilane) at room temperature. After removing the solvent under a stream of nitrogen, the residue was neutralized with 0.1 M NaOH. The solution was extracted with dichloromethane, and the aqueous phase was under nitrogen protection. Saline containing [^99m^TcO_4_]^-^ (1 mL, 15MBq) was added to a kit containing stannous chloride dihydrate(0.05 mg), succinic dihydrazide (SDH, 5.0 mg) and propylenediamine tetraacetic acid (PDTA, 5.0 mg). The mixture was kept at room temperature for 15 min. Next, the MAG-ABPP solution (1 mL) was added and the reaction allowed to stand for 15 min at 100 ºC to give the final complex ^99m^TcN-MAG-ABCPP (**14a**). ^99m^TcN-MAF-ABCPP (**14b**) and ^99m^TcN-MAA-ABCPP (**14c**) were prepared using the same method. Formation of the complexes and nature of the species formed was determined by Radio-HPLC analysis. Solvent system used for elution was water (solvent A) and acetonitrile (solvent B). The HPLC gradient system for analysis of the product started with 100% A/0% B with a linear gradient to 0% A/100% B from 0 to 30 min. The flow rate was 1.0 mL/min. Five microliters of the sample was used for analysis. Recovery was determined by summing the total counts in all fractions and comparing them to the total injected activity.

### 3.4. Measurement of partition coefficients

The partition coefficients of the three complexes were determined according to the published method [16] by mixing each complex with an equal-volume of 1-octanol and phosphate buffer (0.025 M, pH 7.4) in a centrifuge tube. The mixture was vortexed at room temperature for 1 min and then centrifuged at 5,000 rpm for 5 min. From each phase, 0.1 mL of the aliquot was pipetted and counted in a well-type NaI(Tl) detector. The measurement was repeated three times. Care was taken to avoid cross contamination between the phases. Usually the final partition coefficient value was expressed as log P, where the partition coefficient, P, was calculated using the following equation:

P = (cps in octanol-cps in background)/(cps in buffer-cps in background)

### 3.5. In vitro stability study

The stability of the complexes was studied by measuring the radiochemical purity using HPLC analysis at different time intervals after preparation. The complexes were added to test tubes containing PBS solution (0.025 M, pH 7.4). The mixtures were incubated by shaking them at 37 ºC in a thermomixer. The radiochemical purity was measured at 15 min, 30 min, 60 min and 120 min by Radio-HPLC.

### 3.6. Biodistribution study

Biodistribution studies were performed in Kunming female mice (weighing 18–20 g) bearing S 180 tumors, which grew to a leg diameter of 10-15 mm. ^99m^TcN-MAG-ABCPP, ^99m^TcN-MAF-ABCPP and ^99m^TcN-MAA-ABCPP (0.1 mL, 7.4 × 10^5^ Bq) were injected via a tail vein and the injected radioactivity was measured with a well-type NaI(Tl) detector, respectively. The mice were sacrificed at 5 min, 30 min, 60 min and 120 min post-injection. The tumor, other organs of interest and blood were collected, weighed and measured for radioactivity. The results were expressed as the percent uptake of injected dose per gram of tissue (%ID/g). All biodistribution studies were carried out in compliance with the national laws related to the conduct of animal experimentation. 

## 4. Conclusion

In summary, the new 3-cyanopyrazolo[1,5-a]pyrimidine ABCPP was successfully synthesized and conjugated with MAG, MAF and MAA, respectively. The three compounds were labeled with ^99m^Tc in high radiochemical purities through a ligand exchange reaction, which can be easily used for the preparation of a radiopharmaceutical through a freeze-dried kit formulation. The three complexes demonstrated tumor accumulation, high tumor-to-muscle (T/M) ratios and fast clearance from blood and muscle. Among them, ^99m^TcN-MAG-ABCPP showed the most favorable characteristics and could be further studied as potential tumor imaging agent.

## Figures and Tables

**Table 1:** Partition coefficients of ^99m^TcN-MAG-ABCPP, ^99m^TcN-MAF-ABCPP and ^99m^TcN-MAA-ABCPP.

	^99m^TcN-MAG-ABCPP	^99m^TcN-MAF-ABCPP	^99m^TcN-MAA-ABCPP
logP	-1.54 ± 0.02	-1.18 ± 0.02	-1.35 ± 0.01

**Table 2:** Biodistribution of ^99m^TcN-MAG-ABCPP in mice bearing S 180 tumor (%ID/g)^a^.

Tissue	5 min	30 min	60 min	120 min
Heart	0.93 ± 0.11	0.32 ± 0.06	0.35 ± 0.02	0.37 ± 0.08
Liver	23.32 ± 1.32	23.88 ± 2.83	14.67 ± 0.87	12.84 ± 0.91
Spleen	19.90 ± 1.70	14.38 ± 1.64	8.16 ± 0.10	8.32 ± 1.05
Lung	4.53 ± 0.99	2.30 ± 0.60	0.86 ± 0.07	0.73 ± 0.41
Kidney	11.08 ± 1.13	5.06 ± 1.16	3.58 ± 0.21	3.35 ± 0.29
Bone	1.25 ± 0.74	0.95 ± 0.27	0.73 ± 0.28	0.86 ± 0.09
Muscle	0.72 ± 0.05	0.39 ± 0.08	0.34 ± 0.07	0.22 ± 0.03
Stomach	0.69 ± 0.12	0.54 ± 0.11	0.32 ± 0.04	0.09 ± 0.02
Large Intestine	1.62 ± 0.20	0.62 ± 0.08	0.68 ± 0.02	0.67 ± 0.06
Small Intestine	8.82 ± 1.29	3.42 ± 0.42	0.64 ± 0.08	0.62 ± 0.15
Blood	2.38 ± 0.17	0.77 ± 0.01	0.46 ± 0.04	0.38 ±0.01
Brain	0.13 ± 0.01	0.04 ± 0.004	0.08 ± 0.01	0.07 ± 0.01
Tumor	1.23 ± 0.13	1.16 ± 0.14	0.86 ± 0.14	0.45 ± 0.07
T/M ratio	1.71	2.97	2.49	2.01
T/B ratio	0.52	1.51	1.84	1.17

T/M = tumor-to-muscle, T/B = tumor-to-blood; ^a^ All data are the mean percentage (n = 3) of the injected dose of ^99m^TcN-MAG-ABCPP; per gram of tissue, ± the standard deviation of the mean.

**Table 3:** Biodistribution of ^99m^TcN-MAF-ABCPP in mice bearing S 180 tumor (%ID/g)^b^.

Tissue	5 min	30 min	120 min	180 min
Heart	0.82 ± 0.02	0.53 ± 0.09	0.35 ± 0.02	0.29 ± 0.04
Liver	20.12 ± 1.03	15.55 ± 1.33	14.46 ± 0.41	14.42 ± 0.89
Spleen	18.62 ± 1.11	16.39 ± 1.21	3.09 ± 0.41	4.25 ± 0.37
Lung	4.84 ± 0.56	4.11 ± 0.21	1.18 ± 0.25	0.89 ± 0.08
Kidney	10.25 ± 0.92	5.36 ± 0.38	3.42 ± 0.22	2.05 ± 0.30
Bone	1.02 ± 0.09	0.79 ± 0.08	0.62 ± 0.06	0.62 ± 0.07
Muscle	0.68 ± 0.02	0.28 ± 0.04	0.16 ± 0.06	0.17 ± 0.02
Stomach	5.12 ± 0.22	4.65 ± 0.42	2.62 ± 0.33	2.38 ± 0.05
Large Intestine	1.03 ± 0.10	0.33 ± 0.01	0.25 ± 0.03	0.50 ± 0.07
Small Intestine	1.45 ± 0.08	1.39 ± 0.04	0.31 ± 0.08	0.37 ± 0.06
Blood	2.45 ± 0.12	1.44 ± 0.05	0.94 ± 0.07	0.69 ± 0.05
Brain	0.12 ± 0.01	0.06 ± 0.006	0.05 ± 0.007	0.06 ± 0.01
Tumor	0.89 ± 0.07	0.74 ± 0.08	0.41 ± 0.01	0.45 ± 0.02
T/M ratio	1.31	2.56	2.63	2.57
T/B ratio	0.36	0.51	0.44	0.66

T/M = tumor-to-muscle, T/B = tumor-to-blood; ^b^ All data are the mean percentage (n = 3) of the injected dose of ^99m^TcN-MAF-ABCPP; per gram of tissue, ± the standard deviation of the mean.

**Table 4:** Biodistribution of ^99m^TcN-MAA-ABCPP in mice bearing S 180 tumor (%ID/g)^c^.

Tissue	5min	30min	60min	120min
Heart	1.09 ± 0.13	0.43 ± 0.08	0.32 ± 0.005	0.29 ± 0.02
Liver	24.76 ± 1.18	20.46 ± 2.20	22.47 ± 1.73	24.19 ± 3.07
Spleen	15.81 ± 1.73	6.87 ± 1.29	7.31 ± 0.58	7.85 ± 0.76
Lung	8.31 ± 0.59	2.94 ± 0.57	2.30 ± 0.10	2.67 ± 0.34
Kidney	5.58 ± 0.55	2.59 ± 0.24	2.33 ± 0.11	2.08 ± 0.27
Bone	0.89 ± 0.08	1.11 ± 0.13	0.57 ± 0.07	0.50 ± 0.03
Muscle	0.63 ± 0.05	0.34 ± 0.07	0.30 ± 0.08	0.17 ± 0.04
Stomach	3.74 ± 0.51	4.53 ± 0.31	3.43 ± 0.55	2.79 ± 0.41
Large Intestine	0.97 ± 0.16	0.48 ± 0.08	0.33 ± 0.02	0.43 ± 0.06
Small Intestine	5.17 ± 0.39	2.92 ± 0.22	1.79 ± 0.09	1.00 ± 0.14
Blood	2.85 ± 0.15	1.12 ± 0.11	0.74 ± 0.02	0.67 ± 0.07
Brain	0.12 ± 0.01	0.07 ± 0.01	0.05 ± 0.01	0.05 ± 0.008
Tumor	0.92 ± 0.03	0.81 ± 0.05	0.51 ± 0.03	0.42 ± 0.05
T/M ratio	1.47	2.32	1.72	2.47
T/B ratio	0.33	0.72	0.70	0.63

T/M = tumor-to-muscle, T/B = tumor-to-blood; ^c^ All data are the mean percentage (n = 3) of the injected dose of ^99m^TcN-MAA-ABCPP; per gram of tissue, ± the standard deviation of the mean.
